# Prevalence of anaemia and associated factors among preterm infants at six weeks chronological age at Muhimbili National Hospital, Dar es Salaam, Tanzania: a cross-sectional study

**DOI:** 10.11604/pamj.2023.44.193.31190

**Published:** 2023-04-20

**Authors:** Zawadi Edward Kalezi, Rodrick Kisenge, Helga Naburi, Alphonce Nsabi Simbila, Martha Mkony, Germana Leyna

**Affiliations:** 1 Department of Paediatrics and Child Health, Muhimbili University of Health and Allied Sciences, Dar es Salaam, Tanzania,; 2 Emergency Medicine Department, Muhimbili University of Health and Allied Sciences, Dar es Salaam, Tanzania,; 3 Department of Paediatrics and Child Health, Muhimbili National Hospital, Dar es Salaam, Tanzania,; 4 Department of Epidemiology and Biostatistics, Muhimbili University of Health and Allied Sciences, Dar es Salaam, Tanzania

**Keywords:** Preterm infants, phlebotomy, anaemia, Tanzania

## Abstract

**Introduction:**

preterm infants are vulnerable to several medical complications including anaemia, a significant public health problem with consequences on neurodevelopment. This study looked at the magnitude of anaemia and its associated factors among preterm infants at 6 weeks chronological age in a paediatric clinic of Muhimbili National Hospital (MNH).

**Methods:**

this was a hospital based cross-sectional study conducted among preterm infants at 6 weeks chronological age attending follow-up clinic at MNH from October 2019 to March 2020. Parental interviews, medical records reviews and haemoglobin assessment was done during the clinic visits. Logistic regression was used to determine the association between studied factors and anaemia.

**Results:**

the proportion of preterm infants with anaemia at 6 weeks chronological age was 38.4% (142/370) with 74% of these infants having moderate anaemia. Morphological types of anaemia were normocytic (56.3%) and microcytic anaemia (4.9%). Two-thirds of preterm infants (62%) were on haematinics supplementation. Moderate preterm born at gestation age 32 to <34 weeks (OR=2.21, 95% CI 1.15-4.25, p=0.017), two or less phlebotomies (OR=2.3; 95% CI 1.23-4.30; P=0.010) and more than two phlebotomies (OR=7.2, 95% CI 3.62-14.16, p≤0.001) were significantly associated with anaemia.

**Conclusion:**

the proportion of preterm infants with anaemia at 6 weeks chronological age is high despite two-thirds being on haematinics supplementation. Moderate preterm and multiple phlebotomies significantly contributed to the occurrence of anaemia. Screening preterm infants for anaemia, appropriate management, and close follow-up are recommended to reduce its burden.

## Introduction

Globally, around 15 million babies are born prematurely each year with more than 60% of preterm deliveries occurring in Africa and South Asia [1]. Preterm infants face several morbidities such as respiratory distress syndrome (RDS), necrotizing enterocolitis (NEC), neonatal sepsis and anaemia. The prevalence of anaemia varies among studies with a range from 6% to 70% depending on post-natal age [2-6]. In rural South Africa at Moroka Hospital, 9.2% of preterm babies had anaemia before discharge [3] while in Cameroon and Ethiopia the prevalence of anaemia were reported to be 32.9% and 23.2% respectively [7,8]. In Tanzania around 336,000 babies are born prematurely annually which translates to around 17% of all live births [7] and magnitude of anaemia is estimated to be 58% among children aged 6 to 59 months [8].

Factors that contribute to the occurrence of anaemia in preterm infants include lower birth weight and gestation age at birth, rapid increase in body weight, blood loss due to phlebotomy, infections and inadequate nutrition [9,10]. However, impaired erythropoietin (EPO) production is postulated to be the main physiological factor during the first weeks of life at approximately 6 weeks of age [9]. Anaemia may require blood transfusions depending on the severity as well as clinical presentation and if left untreated has negative long-term impact on growth and development particularly the neurobehavior development [11]. World Health Organization (WHO) recommend haematinics supplementation in preterm babies from second week of life so as to improve iron stores and lower the risk of developing iron deficiency anaemia [12-14]. Prophylactic iron supplementation has been reported to reduce the prevalence of anaemia in preterm infants [15].

Despite the high rate of premature delivery in Tanzania, long-term negative impacts of anaemia to growing infants and the fact that premature infants are more vulnerable, the magnitude of anaemia among these infants is not well documented in our setting. Therefore, this study was conducted to determine the magnitude of anaemia among preterm infants at 6 weeks post-natal age as well as associated factors to detect early and prioritise those at risk of developing anaemia.

## Methods

**Study design and setting:** this is a hospital based cross-sectional study conducted at Muhimbili National Hospital (MNH) in Dar es Salaam, Tanzania at the Paediatric Outpatient Department between October 2019 and March 2020.

**Study population:** preterm infants at 6 weeks of chronological age attending follow-up clinic at MNH during the study period were enrolled into the study. However, those with obvious congenital anomalies or who denied consent to participate were excluded. The age group of 6 weeks was chosen in order to capture the true picture of the proportion of the infants with anaemia because the physiologic decrease of haemoglobin content in premature infants is noticed approximately at 6 weeks [9].

**Sample size determination:** the sample size was calculated using Kish Leslie formula, and the estimated minimum sample size was 354. Data collection was done by using a pre-tested researcher administered, standardized structured questionnaire. Data collected included socio-demographic and clinical information for each study participant.

**Study variables:** socio-demographic parameters were gestation age at birth based on last normal menstrual period obtained from mothers' antenatal clinic card and for infant variables including birth weight and gender were obtained from Reproductive and Child Health (RCH) card 1. Maternal variables including maternal age, education level, occupation status and parity were obtained from the interviews.

Clinical parameters were haematinics supplementation, type of feeding, palmar pallor, nutritional status (grouped as normal, underweight, and overweight based on fenton charts), haemoglobin level, mean corpuscular volume (MCV) and mean corpuscular haemoglobin (MCH), blood transfusion and phlebotomy history (assessed by counting the number of times the blood samples had been recorded in the computer system by dates). The frequency of blood investigations recorded was used to estimate the amount of blood that was drawn per investigation. This was established since our hospital central laboratory accepts a minimum of approximately 2 mls per blood test, thus if blood was drawn > 2 times, we estimated blood loss to be a minimum of 4 ml. The anticipated recall bias was mitigated by cross-checking with computer records. The outcome (anaemia) was assessed using a portable haemoglobinometer (HemoCue 301) and a cut-off point was haemoglobin levels below 11g/dl according to WHO guideline. Severity of anaemia was classified as mild anaemia (10.0-10.9g/dl), moderate anaemia (7.0 to 9.9g/dl) and severe anaemia (<7g/dl) [13].

**Sample collection and testing:** a blood sample of approximately 10 microlitre (heel prick) was drawn into the cavity of microcuvette by capillary action and inserted in the haemoglobinometer. Additionally, a sample of one millilitre of venous blood was aseptically drawn from antecubital of each premature infant found to have anaemia and blood was placed into EDTA tube that was properly labelled and immediately sent to the haematology laboratory for full blood picture (FBP) analysis. This was done according to the hospital standard of care and preterm infants who had anaemia were referred to the attending paediatrician immediately for further management.

**Data management and analysis:** data entry and cleaning was done using Statistical Package for Social Science (SPSS) version 25. Description of means, medians, frequencies, and proportions of the given data of each variable was calculated. Contingency tables were constructed for bivariate analysis to explore factors associated with anaemia. To determine associations between dependent and independent variables, Chi-square test and where applicable Fischer's exact test were used for categorical variables while Student's t-test and Mann-Witney U test were used for continuous variables. The level of significant association was set at p <0.05. Binary logistic regression model was used to determine Odds ratios and 95% confidence interval as well as p-values for the factors associated with anaemia. Only factors with p-values <0.2 on univariate analysis were used in multivariate analysis. Adjusted Odds ratios with p-value <0.05 on multivariate analysis were considered significant. For the missing data, case deletion approach was used.

**Ethical approval:** to conduct the study, it was obtained from the Ethics Review Committee of the Muhimbili University of Health and Allied Sciences and MNH (Ref.No.DA.287/298/01A).

## Results

**Figure 1:**
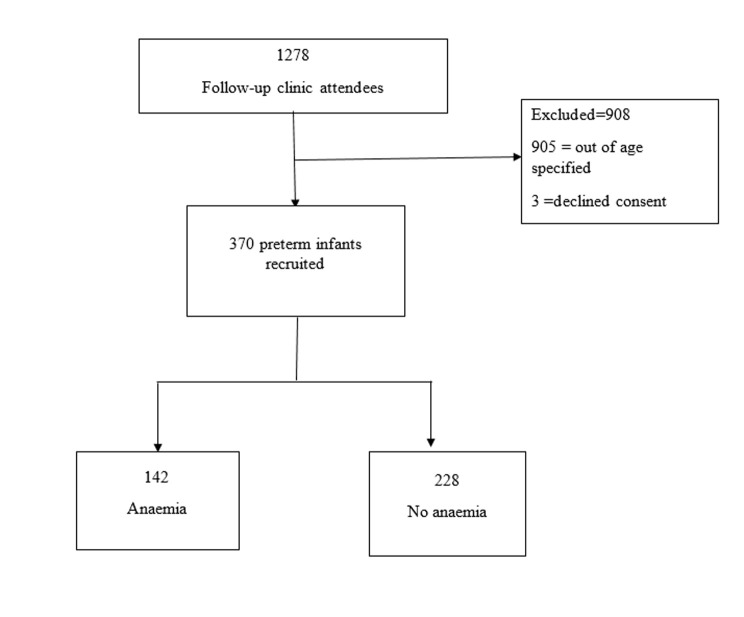
flow diagram showing the recruitment of the study participants and the outcome

There were a total of 1278 premature infants who attended the follow-up clinic during the study period. A total of 370 premature babies met the inclusion criteria and were enrolled into the study and their data were included in the final analysis (Figure 1). Out of 370 preterm infants recruited, 48% of infants were born before 32 weeks of gestation with median gestation age of 32 weeks (IQR= 28-34). Sixty percent of infants weighed more than 1.5kg at birth (Table 1). Thirty-two percent (120/370) of the study participants had received blood transfusion with median blood transfusion frequency being one (IQR 1-2). Nearly three quarter 275 (74%) of the study participants has had blood drawn for investigations at least once, with femoral site being the major puncture site (Table 2).

**Table 1:** maternal and infants socio-demographic characteristics among 370 preterms at MNH, Dar es Salaam, Tanzania

Variable	Category	Frequency (%)
**Infants Characteristics**		
Sex	Male	170 (46)
	Female	200 (54)
Gestation age (weeks)	<32	178 (48)
	32-<34	82 (22)
	≥34	110 (30)
Birth weight (kg)	<1.5	148 (40)
	≥ 1.5	222 (60)
Nutritional status	Normal	188 (51)
	Underweight	173 (47)
	Overweight	9 (2)
**Maternal Characteristics**		
Maternal age (years)	<25	107 (29)
	25-34	194 (52)
	>35	69 (19)
Maternal education	No formal education	13 (4)
	Primary education	196 (53)
	Above primary	161 (43)
Maternal occupational	Employed	52 (14)
	Unemployed	318 (86)
Marital status	Married/ cohabiting	341 (92)
	Single /divorced	29 (8)

**Table 2:** maternal and infant clinical characteristics among 370 preterms at MNH, Dar es Salaam, Tanzania

Variable	Category	Frequency (%)
**Infants’ clinical characteristics**		
Blood transfusion status	Yes	120 (32)
	No	250 (68)
Blood transfusion frequencya	≤ 2	97 (81)
	> 2	23 (19)
Phlebotomy status	None	95 (26)
	≤ 2	170 (46)
	>2	105 (28)
Phlebotomy siteb	Femoral	196 (71)
	Other	79 (29)
Haematinics use status	Yes	228 (62)
	On time	87(38)
	Delayed	141 (62)
	No	142 (38)
Haematinics dosage (mg /kg/day of elemental iron) c	Appropriate dose	29 (13)
	Above dosage	199 (87)
Palmar pallor	No	299 (81)
	Some	39 (10)
	Severe	32 (9)
**Maternal clinical characteristics**		
Maternal haemoglobin level (during pregnancy) (g/dl)	<11	152 (49)
	≥11	156 (51)
	Unknown	62

aOut of 120 preterm infants who had blood transfusion, bOut of 275 preterm infants who had blood investigations, cOut of 228 of those on haematinics

The overall proportion of anaemia among preterm infants at 6 weeks of age was 38.4% (142/370) and majority (74%, 105/142) had moderate anaemia. Morphological type of anaemia was normocytic (56.3%) and microcytic anaemia (4.9%).

The mean birth weight of study participants with anaemia was approximately 0.1 kg lower compared to those without anaemia (p=0.031) on bivariate analysis. Other factors that were significantly associated with anaemia included gestation age at birth and phlebotomy status (Table 3). Preterm infants who were born between 32 weeks of gestation to below 34 weeks had two times higher odds of developing anaemia compared to those who were born ≥ 34 weeks (OR=2.21; 95% CI 1.15-4.25; p=0.017). Compared to those who had no history of phlebotomy, the odds of developing anaemia increased as the number of phlebotomies increased (OR=2.3; 95% CI 1.23-4.30; p=0.010) and (OR=7.2; 95% CI 3.62-14.16; p=<0.001) (Table 4).

**Table 3:** factors associated with anaemia among 370 preterms at MNH, Dar es Salaam, Tanzania

Variable	Category	Anaemia		P-value (Chi2)
		Yes	No	
**Birth weight (kg)**	Mean ± SD	1.53 ± 0.39	1.62 ± 0.40	0.029a
**Size for gestation age**	SGA	33 (39%)	51 (61%)	0.93
	AGA	80 (38%)	133 (62%)	
	LGA	29 (40%)	44 (60%)	
**Gestation age**	< 32	69 (39%)	109 (61%)	0.005
	32-<34	42 (51%)	40 (49%)	
	≥34	31 (28%)	79 (72%)	
**Maternal education**	None/primary	85 (41%)	124 (59%)	0.30
	Above primary	57 (35%)	104 (65%)	
**Maternal age**	Mean ± SD	28.59 ± 6.51	28.34 ± 5.89	0.70a
**Parity**	Median (IQR)	2 (1-3)	2 (1-3)	0.67b
**Phlebotomy status**	None	18 (19%)	77 (81%)	<0.001c
	≤ 2	58 (34%)	112 (66%)	
	> 2	66 (63%)	39 (37%)	
**Blood transfusion**	Yes	49 (41%)	71 (59%)	0.50
	No	93 (37%)	157 (63%)	
**Haematinics use**	Yes	85 (37%)	143 (63%)	0.59
	No	57 (40%)	85 (60%)	
**Haematinics dosage**	Appropriate	12 (41%)	17 (59%)	0.63*
	Above dosage	73 (37%)	126 (63%)	
**Exclusive breastfeeding**	Yes	138 (38%)	227 (62%)	0.07d
	No (formula fed)	4 (80%)	1 (20%)	

aStudent t-test, bMann-Whitney U test, cChi-square test for trend, dFischer’s exact test, *Out of 228 of those on haematinics. IQR, interquartile range; SD, standard deviation; SGA, small for gestation age; AGA, appropriate for gestation age; LGA, large for gestation age.

**Table 4:** independent factors associated with anaemia among 370 preterms at MNH, Dar es Salaam, Tanzania

Variable	Univariate analysis		Multivariate analysis	
	cOR (95% CI)	P-value	aOR (95% CI)	P-value
Birth weight	1.80 (1.06-3.08)	0.030	0.81 (0.41-1.61)	0.55
**Size for gestation age**				
AGA	1			
SGA	1.08 (0.64-1.81)	0.78		
LGA	1.10 (0.64-1.89)	0.74		
**Gestation age**				
<32	1.61 (0.97-2.70)	0.07	1.05 (0.56-1.98)	0.87
32-<34	2.68 (1.47-4.88)	0.001	2.21 (1.15-1.98)	0.017
≥34	1			
**Maternal education**				
None /primary	1			
Above primary	1.25 (0.82-1.91)	0.30		
**Maternal age**	0.99 (0.96-1.03)	0.69		
**Parity**	0.94 (0.81-1.10)	0.44		
**Phlebotomy status**				
None	1		1	
≤ 2	2.22 (1.21-4.05)	0.010	2.30 (1.23-4.30)	0.010
>2	7.24 (3.79-13.84)	<0.001	7.16 (3.62-14.16)	<0.00
**Blood transfusion**				
No	1			
Yes	1.17 (0.75-1.82)	0.50		
**Haematinics use**				
No	1			
Yes	0.89 (0.58-1.36)	0.58		
**Exclusive breastfeeding**				
No				
Yes	0.15 (0.02-1.37)	0.09	0.12 (0.01-1.10)	0.06

cOR, crude odds ratio; aOR, adjusted odds ratio; CI, confidence interval; SGA, small for gestation age; AGA, appropriate for gestation age; LGA, large for gestation age.

## Discussion

In this study, we found a high proportion of preterm infants (38%) with anaemia; with nearly three-quarters having moderate anaemia. Anaemia was prevalent among moderate preterm infants (born at gestation age 32 to <34 weeks) and those with multiple phlebotomies. Birth weight, maternal age, parity and haematinics did not influence the occurrence of anaemia in these infants. A high burden of anaemia found in this study could be due to the physiological post-natal haemoglobin drop which occurs in new-borns during the first weeks of life and reaches nadir level at approximately 4-6 weeks in preterm infants. However, other studies in Brazil [2], India [14], South Africa [3], Cameroon [7] and Ethiopia [6] reported lower proportions. This disparity of proportions across studies may partly be explained by the differences in the age of the study participants.

In addition, other studies have reported much higher proportions, for instance, 58% in Cardiff, Wales [4] among very preterm infants and 70% in Indonesia [5] among 2 months old preterm infants. Of note, the study in Indonesia was conducted around the time when a high rate of post-natal catchup growth occurs. Moreover, a small sample in the study done in Indonesia might have over-exaggerate the prevalence. Despite the varying magnitude of anaemia among preterm babies in different contexts, the screening for preterm infants should be emphasized to capture those with problems early and provide effective treatment to prevent poor neurodevelopmental outcomes.

Among the factors studied, multiple phlebotomies were associated with the occurrence of anaemia in the current study. These findings are in keeping with what has been reported by a study done in Iowa, U.S.A [15-17] where by blood loss equivalent to 2 to 4 ml/kg/week contributed to anaemia in preterm infants. This is also consistent with findings by Widness [10] in which anaemia was observed among preterm infants with phlebotomy blood loss of 11 to 22ml/kg/week in the intensive care unit in their first 6 weeks of life. Similarly, several other studies reported blood loss as a result of blood testing as among the primary contributors to the development of anaemia in preterm infants [18-20]. These findings imply that; it is important to reduce phlebotomy blood loss using strategies such as micro-sampling and ordering only necessary investigations in preterm infants especially during early postnatal life.

We also found higher proportion of anaemia among moderate preterm infants compared to late preterm infants. These findings were similarly reported by other studies done in Cameroon [7], China [16] and Wales [4]. This is probably a reflection of the fact that haemoglobin increases with advancing gestation age, thus babies who are born before the 3^rd^ trimester of gestation are deprived of most of the iron transported from the mother and a great deal of in utero foetal erythropoiesis.

In our study, more than half of the preterm infants were on haematinics supplementation. This is contrary to what has been found by Kasasa *et al*. [13] years ago which may be explained by currently available structured follow-up clinic in our hospital which was not the case then. Furthermore, Iron supplementation has been recommended by the WHO for VLBW infants starting at second week of life to improve their iron stores and lower the risk of developing iron deficiency anaemia [12]. Similarly, a study done in China has also reported that prophylactic iron supplementation to their preterm infants reduce the prevalence of anaemia [15,21]. Screening for anaemia in this study was done using HemoCue 301 analyser which is a reliable diagnostic stool and uses minimal amount of blood.

There were few limitations; like inability to assess comorbid conditions such as sepsis as one of the contributing factors for anaemia both related to its effect through hemolysis and necessity for frequent blood work ups. Moreover, it was difficult to quantify the exact amount of blood loss due to phlebotomy, instead number of blood draws was used as a proxy. Despite the limitations, the study opens our eyes on the burden of anaemia in these children.

## Conclusion

Burden of anaemia among preterm infant at our setting is high with majority presenting with moderate anaemia. Multiple phlebotomies and moderate preterm were significantly associated with anaemia. Screening of the preterm infants especially those at risk should be emphasized. Further, minimizing the number of phlebotomies will be invaluable for preventing anaemia in preterm infants.

### What is known about this topic

- *Anaemia is associated with delayed neurodevelopment in preterm infants;*
- *Anaemia is a significant public health problem in children;*
- *Anaemia is among the complications of prematurity*.

### What this study adds

- *The magnitude of the anaemia in preterm infants at our setting is high;*
- *Multiple phlebotomies and moderate gestation age are associated with anaemia in preterm infants*.
